# Supplementary material for: Applying GRADE-CERQual to qualitative evidence synthesis findings—paper 2: how to make an overall CERQual assessment of confidence and create a Summary of Qualitative Findings table
Source: Implement Sci. 2018 Jan 25;13(Suppl 1):10. doi: 10.1186/s13012-017-0689-2 (PMC5791047; doi:10.1186/s13012-017-0689-2)
Supplement: Supplementary file 2 — Deciding whether to apply CERQual to a review finding—interpretive or explanatory level findings. (PDF 637 kb) [file 13012_2017_689_MOESM2_ESM.pdf]

Additional file 2. Deciding whether to apply CERQual to a review finding – interpretive or explanatory level findings

Consider the following more explanatory review finding, which brings together five descriptive findings from a qualitative evidence synthesis of factors affecting the implementation of lay (or community) health worker programmes (adapted from [1]):

> *A review of factors affecting the implementation of lay health worker programmes found that where these programmes are closely integrated into the health system (finding 1 – low confidence) and where individual lay health workers and health professionals work together closely (finding 2 – high confidence), good lay health worker-professional relationships may develop. The development of good working relationships may be negatively affected where health professionals are concerned that lay health workers may be overconfident and not sufficiently skilled (finding 3 – low confidence). However, the development of good working relations may be more likely where interactions are respectful, supportive and egalitarian (finding 4 – low confidence); and also where professionals see lay health workers as likely to decrease their workload and bring supplementary skills and knowledge (finding 5 – moderate confidence).*

This review finding attempts to explain how good lay health worker-professional relationships may develop and is therefore explanatory or interpretive in nature. The explanation is based on a number of more descriptive review findings from the same review, which vary from low to high in their CERQual level of confidence assessments. This broad finding also links the descriptive findings in a pattern that attempts to create a logical explanation. Linking the individual descriptive findings in this pattern may be more or less supported by the available data. In other words, the review author may have hypothesised some of the linkages as part of the interpretive process rather than basing them on linkages described in the primary studies.

The review team has two options in relation to assessing confidence in this review finding:

- To not attempt to assess overall confidence in the explanatory finding. This is based on the rationale that each of the contributing descriptive findings is based on different studies and data, making it challenging to assess overall confidence
- To attempt to assess overall confidence in the explanatory finding. This could be done by looking at the CERQual assessments for contributing descriptive findings, including their underlying component assessments, and looking at the evidence supporting the links between the contributing descriptive findings, and attempting to make an assessment for the broad explanatory finding. At present we do not have experience in, or guidance for, undertaking CERQual assessments of this kind. This is part of the research agenda for CERQual

Some interpretive or explanatory findings may emerge directly from the analysis process rather than through bringing together multiple, more descriptive findings as in the example above. CERQual can be applied in the usual way to interpretive findings that emerge directly from the analysis process.

References

1. Glenton C, Colvin CJ, Carlsen B, Swartz A, Lewin S, Noyes J, Rashidian A: **Barriers and facilitators to the implementation of lay health worker programmes to improve access to maternal and child health: qualitative evidence synthesis**. *The Cochrane database of systematic reviews* 2013, **10**:CD010414.
