# Supplementary material for: Applying GRADE-CERQual to qualitative evidence synthesis findings—paper 2: how to make an overall CERQual assessment of confidence and create a Summary of Qualitative Findings table
Source: Implement Sci. 2018 Jan 25;13(Suppl 1):10. doi: 10.1186/s13012-017-0689-2 (PMC5791047; doi:10.1186/s13012-017-0689-2)
Supplement: Supplementary file 3 — Guidance on applying CERQual to a qualitative evidence synthesis conducted by another review team. (PDF 650 kb) [file 13012_2017_689_MOESM3_ESM.pdf]

Additional file 3. Guidance on applying CERQual to a qualitative evidence synthesis conducted by another review team

CERQual should only be applied to well conducted reviews. The following questions may help when assessing how well a review was conducted [1]:

1. Did the review address an appropriate question?
2. Was a clear and appropriate explanation provided for the search approach used?
3. Were the criteria used to select studies appropriate?
4. Was the approach used to appraise the methodological limitations of the included studies appropriate?
5. Was an appropriate approach used to analyse the findings of the included studies?

If your assessment is that the review was not well conducted overall, you should probably not apply CERQual, as this will not give useful results.

Applying CERQual to the findings of a well conducted qualitative evidence synthesis undertaken by others:

If the answer to any of the following questions is 'no', it may be very difficult to apply the CERQual approach:

1. Does the synthesis include a detailed description of its main findings?
2. If the synthesis used a sampling approach to include primary studies, is this approach described transparently?
3. Does the synthesis indicate which primary studies contributed to each review finding?
4. Does the synthesis include a 'Characteristics of included studies' table (or similar) that reports the following information for each included primary study: where the study was conducted (country, setting); study participants; sampling approach used; data collection and analysis methods used?
5. Did the review team assess the methodological limitations of the studies included in the synthesis using a standard tool (e.g. CASP for qualitative studies [2]) and are the assessments for each study (including for each element of the tool) reported in the synthesis?*

Process:

- Identify all of the reports or papers reporting findings from the synthesis. Findings may be reported in more than one paper for some syntheses
- Identify the main review findings in the synthesis and write an initial summary of review findings for these
- For each review finding, list the primary studies that contributed to the finding and summarise their context/s
- Create an evidence profile template for each finding
- Apply CERQual to each finding, noting where your assessment of a particular component is limited by the information available in the published synthesis. Where your assessment of a specific component is limited in this way, a 'conservative' approach should be taken by assuming that there were moderate concerns regarding this component
- Insert each CERQual component assessment into the relevant evidence profile and make an overall assessment for each CERQual finding. Again, note in your explanation of the CERQual assessment where this is limited by the information available in the published synthesis
- Create a Summary of Qualitative Findings (SoQF) table

Specific pointers:

- Methodological limitations: CERQual does not recommend the use of a specific assessment tool and those applying CERQual need to judge if the tool used in a synthesis was appropriate. Some syntheses may present only an overall 'methodological quality' score for each included study. In these cases, the limitations of this for the CERQual assessment need to be acknowledged. In addition, some assessment tools include items related to adequacy and relevance. In these cases, care should be taken not to downgrade findings twice for the same concerns
- Relevance: as noted above, it is not possible to assess this component if a synthesis does not include a 'Characteristics of included studies' table as insufficient detail or missing characteristics may impair the quality of the relevance judgements. CERQual cannot be applied to such syntheses without going back to the included primary studies
- Coherence: unless a synthesis presents detailed tables of the data contributing to each review finding, it may not be possible to assess this component
- Adequacy of data: for many syntheses, adequacy may need to be assessed based on only the number of studies contributing data to a review finding and not the depth of the data. This limitation needs to be acknowledged
- Where some of the information needed to make a CERQual assessment is not available in the published synthesis, you could consider contacting members of the review team for further information or obtaining the relevant primary studies. We acknowledge, however, that it may generally not be feasible to undertake either of these tasks. It may also be helpful to send the draft SoQF table, including the CERQual assessments, to the review team for comment.

**It is possible to assess the methodological limitations of the included primary studies post hoc, but this would involve substantially more work requiring examination of the full text for each primary study.*

References

1. Lewin S, Bosch-Capblanch X, Oliver S, Akl EA, Vist GE, al. e: **Guidance for Evidence-Informed Policies about Health Systems: Assessing How Much Confidence to Place in the Research Evidence**. *PLOS Medicine* 2012, **9**(3):e1001187.
2. CASP: **Qualitative Appraisal Checklist for Qualitative Research**. Available at: http://www.casp-uk.net/#!casp-tools-checklists/c18f8: Critical Appraisal Skills Programme; 2011.
