# Supplementary material for: Applying GRADE-CERQual to qualitative evidence synthesis findings—paper 2: how to make an overall CERQual assessment of confidence and create a Summary of Qualitative Findings table
Source: Implement Sci. 2018 Jan 25;13(Suppl 1):10. doi: 10.1186/s13012-017-0689-2 (PMC5791047; doi:10.1186/s13012-017-0689-2)
Supplement: Supplementary file 4 — Recommended standard phrases for describing the assessment for each CERQual component and the overall assessment. (PDF 598 kb) [file 13012_2017_689_MOESM4_ESM.pdf]

Additional file 4. Recommended standard phrases for describing the assessment for each CERQual component and the overall assessment

Assessments for each CERQual component

For each CERQual component, the assessment should be described as follows:

- No or very minor concerns regarding methodological limitations / relevance / coherence / adequacy
- Minor concerns regarding methodological limitations / relevance / coherence / adequacy [*concerns to be described*]
- Moderate concerns regarding methodological limitations / relevance / coherence / adequacy [*concerns to be described*]
- Serious concerns regarding methodological limitations / relevance / coherence / adequacy [*concerns to be described*]

Descriptions of the assessment for each component can include standard descriptors of the nature of the concerns identified, and we encourage users of CERQual to include these so as to improve the transparency of assessments. Here are some examples:

- Moderate concerns regarding relevance as the data contributing to the review finding were *partially relevant*. The data were from primary care settings only while the review question addresses primary and secondary care settings
- Serious concerns regarding relevance as the relevance of the data contributing to the review finding was *unclear*. The studies did not include information on whether the data were collected in primary or secondary care settings
- Serious concerns regarding adequacy as the *quantity of data* was limited, with only two studies, including a total of nine participants, contributing to the review finding
- Minor concerns regarding adequacy as the *richness of data* was generally good, but the one study from Africa provided very little detail regarding how participants accessed care

We recommend also noting where there are no or very minor concerns about a component, as follows: 'There were no or very minor concerns regarding methodological limitations'. No further explanation is required.

Overall CERQual assessment

The overall assessment should be described as follows:

- High confidence
- Moderate / Low / Very low confidence due to [*minor / moderate / serious*] concerns about [*component 1*]; [minor / moderate / serious] concerns about [*component 2*]; etc.

Where a review finding is assessed as being 'high confidence', no further explanation is required as the starting point of 'high confidence' reflects a view that each review finding should be seen as a reasonable representation of the phenomenon of interest unless there are factors that would weaken this assumption.
